# Facile Surfactant‐Free Synthesis of p‐Type SnSe Nanoplates with Exceptional Thermoelectric Power Factors

**DOI:** 10.1002/anie.201601420

**Published:** 2016-04-20

**Authors:** Guang Han, Srinivas R. Popuri, Heather F. Greer, Jan‐Willem G. Bos, Wuzong Zhou, Andrew R. Knox, Andrea Montecucco, Jonathan Siviter, Elena A. Man, Martin Macauley, Douglas J. Paul, Wen‐guang Li, Manosh C. Paul, Min Gao, Tracy Sweet, Robert Freer, Feridoon Azough, Hasan Baig, Nazmi Sellami, Tapas K. Mallick, Duncan H. Gregory

**Affiliations:** ^1^ WestCHEMSchool of ChemistryUniversity of GlasgowGlasgowG12 8QQUK; ^2^ Institute of Chemical Sciences and Centre for Advanced Energy Storage & RecoverySchool of Engineering & Physical SciencesHeriot-Watt UniversityEdinburghEH14 4ASUK; ^3^ EaStCHEMSchool of ChemistryUniversity of St AndrewsSt Andrews, FifeKY16 9STUK; ^4^ School of EngineeringUniversity of GlasgowGlasgowG12 8QQUK; ^5^ School of EngineeringCardiff UniversityCardiffCF24 3AAUK; ^6^ Materials Science CentreSchool of MaterialsUniversity of ManchesterManchesterM13 9PLUK; ^7^ Environment and Sustainability InstituteUniversity of ExeterPenryn CampusPenrynTR10 9FEUK

**Keywords:** nanomaterials, structures, synthesis, thermoelectrics, tin selenide

## Abstract

A surfactant‐free solution methodology, simply using water as a solvent, has been developed for the straightforward synthesis of single‐phase orthorhombic SnSe nanoplates in gram quantities. Individual nanoplates are composed of {100} surfaces with {011} edge facets. Hot‐pressed nanostructured compacts (*E*_g_≈0.85 eV) exhibit excellent electrical conductivity and thermoelectric power factors (*S* ^2^ *σ)* at 550 K. *S* ^2^ *σ* values are 8‐fold higher than equivalent materials prepared using citric acid as a structure‐directing agent, and electrical properties are comparable to the best‐performing, extrinsically doped p‐type polycrystalline tin selenides. The method offers an energy‐efficient, rapid route to p‐type SnSe nanostructures.

Growing global energy demands, together with the negative impacts resulting from combustion of fossil fuels, have diverted attention to technologies for sustainable energy generation and conversion.^1^ Thermoelectrics realize direct inter‐conversion between thermal and electrical energy and provide opportunities to harvest useful electricity from waste heat (and conversely to perform refrigeration). The thermoelectric conversion efficiency of a material is determined by its dimensionless figure of merit, *Z* *T*=*S^2^σT*/*κ*, where *S*, *σ*, *T*, and *κ* represent the Seebeck coefficient, electrical conductivity, absolute temperature, and thermal conductivity, respectively.^2^ Extensive efforts have been devoted to the improvement of the thermoelectric performance of state‐of‐the‐art materials,^3^ and to the discovery of new thermoelectrics^4^ with *Z* *T* values >2. Single‐crystalline SnSe combines a high *Z* *T* with a relatively low toxicity and high Earth‐abundance of the component elements.^4^ SnSe crystals possess very low thermal conductivity owing to lattice anharmocity, yielding record high *Z* *T* values of 2.6 and 2.3 at 923 K along the *b* and *c* crystallographic directions, respectively.^4^ Polycrystalline SnSe materials have been fabricated to improve mechanical properties,^5^ but *Z* *T* has been limited to 1, owing to both increased electrical resistivity and thermal conductivity.^5^ Unfortunately, the synthesis of SnSe is protracted and energy‐intensive, involving heating, melting, and annealing at high temperatures (≈800–1223 K).^4^, ^5^ Before the potential of SnSe can be realized, a fast, cost‐effective, and large‐scale synthesis route to the pure selenide that does not sacrifice performance is essential.

Nanostructuring very effectively enhances *Z* *T*. The high density of interfaces improves phonon scattering, decreasing the lattice thermal conductivity.^2^, ^3^ Bottom‐up solution synthesis methods facilitate control of size, morphology, crystal structure, and defects.^6^ However, the organic surfactants that can control morphology through surface modification are commonly electrically insulating, which can drastically reduce the electrical conductivity of the materials.^7^ Ligand replacement methods switch smaller species for long chain surfactant molecules,^7^ but sometimes involve using high toxicity chemicals,^8^ and introduce impurities,7b which again can adversely influence the transport behavior of the materials.7b Organic contamination can be prevented only if a suitable surfactant‐free synthesis strategy can be found,^9^ and to date solution syntheses of SnSe nanostructures have required organic surfactants and/or solvents, for example, oleyamine, trioctylphosphine selenide, and bis[bis(trimethylsilyl) amino]tin(II), while only yielding milligram quantities of materials.^10^ In this study, we demonstrate a surfactant‐free aqueous solution approach towards the preparation of >10 g of SnSe nanoplates, by boiling a mixture of NaHSe and Na_2_SnO_2_ solutions for 2 h. The phase‐pure nanoplates can be hot pressed into dense pellets with outstanding thermoelectric power factors (Scheme 1).

**Scheme 1:**
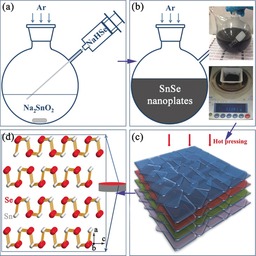
The solution synthesis and hot pressing of SnSe nanoplates: a) injection of NaHSe_(aq)_ into Na_2_SnO_2(aq)_ to trigger the reaction; b) formation of SnSe nanoplates; c) orientation of nanoplates induced by hot pressing; d) structure model of fabricated bulk pellets. The insets in (b) show the nanoplate solution and the yield (≈94 %) from a 2 h synthesis.

Injecting NaHSe into a Na_2_SnO_2_ solution leads to the instant precipitation of SnSe nanoparticles [Supporting Information, Figure S1; Eq. (1)]:

(1) $$\mathrm{NaHSe}+\mathrm{Na}{}_{2}\mathrm{SnO}{}_{2}+H{}_{2}O\to \mathrm{SnSe}+3\;\mathrm{NaOH}$$

Boiling the suspension for 2 h leads to the formation of crystalline, phase‐pure nanoplates of orthorhombic SnSe (ICDD card No. 48‐1224).^11^ Rietveld refinement against powder X‐ray diffraction (PXD) data (Figure 1 a; Tables S1, S2) confirmed the orthorhombic structure (space group *Pnma*, *a=*11.5156(5), *b=*4.1571(2), *c=*4.4302(3) Å). Scanning electron microscopy (SEM; Figures 1 b, S2a) revealed that the product is comprised of rectangular nanoplates, each with lateral dimensions of 80–200 nm and a thickness of 10–60 nm. Energy dispersive X‐ray spectroscopy (EDS; Figure S2b) consistently generated Sn:Se ratios of 49(1):51(1) atom %, while Fourier transform infrared (FTIR) spectra (Figure S3) provided no evidence for organic functional groups (as might be expected from equivalent surfactant‐assisted syntheses).

**Figure 1:**
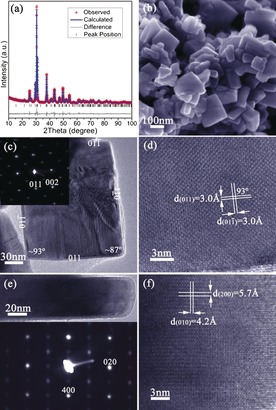
Characterization of SnSe nanoplates synthesized after 2 h: a) profile plot from Rietveld refinement; b) SEM image; c) TEM image of a SnSe nanoplate and its corresponding SAED pattern along the [100] zone axis; d) HRTEM image of part of the plate shown in (c) with d‐spacings indicated; e) profile HRTEM image of a SnSe nanoplate and its corresponding SAED pattern along the [001] zone axis; and f) HRTEM image of part of the plate shown in (e) with d‐spacings indicated.

Transmission electron microscopy (TEM; Figure 1 c) showed that the SnSe nanoplates were almost uniformly rectangular, and selected area electron diffraction (SAED) patterns obtained with the incident electron beam normal to the face of the nanoplate could be indexed along the [100] SnSe zone axis. A set of lattice spacings of ≈3.0 Å intersecting with an angle of 93(1)° could be measured from high resolution TEM (HRTEM; Figure 1 d) corresponding to the {011} plane spacings. Combined with SAED data, the nanoplate face can thus be identified as the *bc* plane of SnSe and the side facets are defined by {011} planes (Figures 1 c, S4). The observed splitting in diffraction spots suggested twin defects induced by orthorhombic distortion.^12^ Images and SAED patterns along the [001] zone axis (beam direction parallel to the nanoplate face; Figure 1 e) verified that: i) the plates are approximately an order of magnitude thinner in the third dimension, and ii) the *bc* plane forms the nanoplate faces. Further, diffraction spots are elongated along [100], indicating planar defects along the *a* axis.^13^ Lattice spacings of ≈5.7 Å (*d*_(200)_) and 4.2 Å (*d*_(010)_) were observed in the corresponding HRTEM image (Figure 1 f).

Intermediate products synthesized after only 1 min of heating were investigated to understand the morphological evolution. The product is single‐phase orthorhombic SnSe (Figure S5a) composed principally of irregular, near‐rectangular nanoplates, many of which are truncated (Figure S5b). TEM revealed internal angles of 133(1)° and 94(1)° at the truncated and regular corners, respectively (Figure 2 a). When correlating the SAED pattern (along the [100] zone axis; Figure 2 b), the TEM image and the crystal plane intersection angle along the *bc* plane (Figure S4) the facets of the SnSe truncated nanoplate can be depicted as shown in Figure 2 a. Hence, the SnSe truncated nanoplate is enclosed by {100} and {011}, together with {001} facets. Given that no surfactant is used, the nanoplate shape is determined primarily by the intrinsic features of the anisotropic selenide crystal structure. Atomic planes with high surface energies usually exhibit fast growth rates, and in SnSe the {001} and {010} planes possess much higher surface energies than the {011} planes.^14^ The former planes would thus experience faster initial growth than the {011} planes. To maintain the minimum surface energy as growth progresses, the {001} and {010} planes diminish, while the {011} planes feature increasingly in the side facets (Figures 2 a,c) until they dominate completely (Figures 1 c, 2 d). The NaOH concentration is also important in regulating growth, and by decreasing the molar ratio from 15:1 to 15:2 the mean length/width of the SnSe nanoplates is reduced from ≈150 nm to ≈80 nm (Figures S6, S7). Decreasing the hydroxide concentration further has more profound effects on the reaction chemistry (see the Supporting Information).

**Figure 2:**
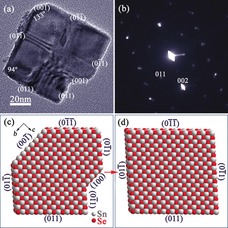
Characterization of SnSe nanostructures synthesized after 1 min: a, b) TEM image and corresponding SAED pattern along the [100] zone axis of a SnSe truncated nanoplate; and c, d) structure models of individual SnSe nanoplates with and without truncation, respectively, established on the basis of the detailed TEM characterization.

The ability to prepare >10 g surfactant‐free SnSe nanomaterials allowed the fabrication of high‐density pellets through hot pressing without the necessity of high temperature annealing. Pellets of ≈95 % theoretical density, retaining the orthorhombic SnSe structure were obtained (denoted **1**; Figure 3 a; Tables S3, S4). Strong orientation of the plates in the *bc* plane is evidenced by the increased intensity of the (*h*00) PXD reflections, and the decrease in peak half‐widths indicates a larger crystallite size after hot pressing. The indirect (direct) optical band gap from diffuse reflectance (DR) UV/Vis spectra10c narrows slightly from ≈0.89 (≈1.1) eV to ≈0.85 (≈1.0) eV (Figure S10) when the nanoplates are consolidated into dense pellets, which could be related to sintering effects. The values are very similar to the indirect band gaps reported for both single crystalline and polycrystalline SnSe.^4^, 5c, 5d **1** is composed of densely packed particles, typically ≈200 nm across with flat surfaces (Figures 3 b, S11a). The Sn:Se ratio remains at 49(1):51(1) atom % (Figure S11b). An SAED pattern (Figure 3 c), with the beam normal to the face of a nanoplate taken from **1** was indexed along the SnSe [100] zone axis. The single‐crystal structure was confirmed by the HRTEM image (Figure 3 d). TEM also showed that the nanoplate from **1** consisted of compacted smaller platelets (Figure S11c). Thermogravimetric analysis (TGA) of **1** under both argon and air revealed negligible weight changes below 500 °C, but suggested that thermal decomposition and oxidation, respectively, begin above this temperature (Figure S12).

**Figure 3:**
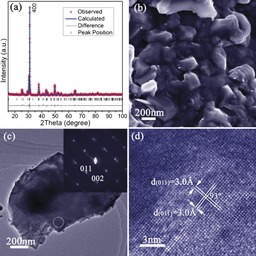
Characterization of SnSe pellet **1**: a) profile plot for **1** from Rietveld refinement against PXD data; b) SEM image of the surface of **1**; c) TEM image of a SnSe peeled nanoplate and its corresponding SAED pattern along the [100] zone axis from the circled area; d) HRTEM image of part of the plate shown in (c) with d‐spacings indicated.

For comparison, a second sample of SnSe nanoparticles (≈40–60 nm) were synthesized by a citric‐acid‐assisted solution synthesis, which were also consolidated into dense pellets (≈92 % of the theoretical density) by hot pressing (denoted **2**; Figures S13, S14). Compared to **1**, **2** possesses the same orthorhombic structure, a similar optical band gap and forms comparable nanostructures (≈200 nm across oriented in the *bc* plane). Importantly, however, Cl is detected in **2** (Sn:Se:Cl ratios of 51(1):48(1):1(1) atom %) that likely originates from the replacement of ligated citric acid by Cl during processing.7b The similar densities and constituent particle sizes of **1** and **2** allowed for a good comparison of their relative electrical performance. The electrical conductivity of **1** (Figure 4 a) increases four‐fold from ≈840 S m^−1^ at 300 K to ≈3500 S m^−1^ at 550 K. The magnitude of the values for **1** can be attributed to the high crystallinity, small band gap, surfactant‐free particle surface, microstructural orientation, and the high level of sintering and densification achieved. By contrast, **2** exhibited electrical conductivity increasing from ≈55 S m^−1^ at 300 K to only ≈250 S m^−1^ at 550 K; more than an order of magnitude lower than **1**.

**Figure 4:**
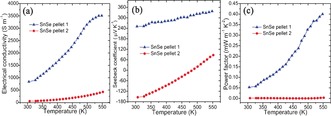
Electrical properties of SnSe pellets **1** and **2** measured perpendicular to the hot pressing direction: a) the electrical conductivity (*σ*), b) the Seebeck coefficient (*S*), and c) the power factor (*S^2^σ*) as a function of temperature.

The contrast in the variation in the Seebeck coefficient with temperature for **1** and **2** is striking (Figure 4 b). *S* for **1** increases almost linearly with temperature (250 μV K^−1^ at room temperature to 340 μV K^−1^ at 550 K). By comparison, **2** shows n‐type behavior at room temperature (*S*≈−150 μV K^−1^), with the value of *S* becoming positive (p‐type behaviour) at ≈490 K and rising to ≈80 μV K^−1^ at 550 K. It is possible that the n‐type conducting behavior correlates to the presence of Cl and/or a slight excess of Sn, as noted above. We are currently investigating this behavior further in systematic doping experiments. An n/p or p/n inversion with increasing temperature has also been observed in pellets consolidated from PbTe, Ag_2_Te, and PbTe_0.1_Se_0.4_S_0.5_ synthesized through surfactant‐assisted solution methods,^7^, ^15^ and should be related to the thermal activation of higher concentrations of positive or negative charge carriers, respectively.7b It is also notable that both *σ* and *S* increase with temperature for **1**. This phenomenon has been observed in both un‐doped and iodine‐doped polycrystalline SnSe.5c,5d, 16 Although the origins of the behavior for **1** require further investigation, the combination of superior *σ* values coupled with high values of *S* leads to exceptional power factors (≈0.05 mW m^−1^ K^−2^ at 300 K to ≈0.40 mW m^−1^ K^−2^ at 550 K; Figure 4 c). In contrast, the power factors for **2** are much lower, (0.001 mW m^−1^ K^−2^ at 300 K and reaching only 0.05 mW m^−1^ K^−2^ at 550 K). The huge differences in performance between **1** and **2** further emphasize the importance of the surfactant‐free synthesis route, not just in the context of a simpler, more sustainable synthesis method, but also in delivering significantly improved electrical properties consistently (Figure S15). Notably, the power factors for **1** far exceed those for un‐doped polycrystalline SnSe across a similar temperature range (0.028–0.04 mW m^−1^ K^−2^),5c–5e and are comparable to those for hole‐doped materials with high carrier concentrations.5d, 17 Recent Na‐ and Ag‐doping studies have elegantly demonstrated how the electrical performance and *Z* *T* values of SnSe single crystals can be dramatically improved.^18^ Given that the samples in our studies were non‐optimized, strategies involving systematic hole doping, in conjunction with surfactant‐free nanostructuring approaches, should yield even higher performing p‐type SnSe materials and pave the way for one‐pot synthesis of p‐ and n‐type SnSe nanomaterials.

In summary, a simple, quick, surfactant‐free, and energy‐efficient solution synthesis yielded SnSe nanoplates in gram quantities. The ensuing nanostructured pellets exhibited exceptional electrical conductivity coupled with high Seebeck coefficients, leading to power factors surpassing those of polycrystalline and surfactant‐coated counterparts. The technique should be readily adaptable to include dopants and amenable to the discovery of further materials, both p‐ and n‐type, with enhanced thermoelectric properties.

## Experimental Section

Full experimental details are provided in the Supporting Information.

Materials Synthesis. 100 mmol NaOH and 10 mmol SnCl_2_⋅2 H_2_O were added into 50 mL deionized water to yield a transparent Na_2_SnO_2_ solution. 50 mL of NaHSe_(aq)_ prepared from Se and NaBH_4_ was injected into the boiling solution, leading to the immediate formation of a black precipitate. The mixture was boiled for 2 h, and cooled to room temperature under Ar_(g)_ on a Schlenk line. The products were washed with deionized water and ethanol and dried at 50 °C for 12 h. Scaled‐up syntheses were performed with six‐fold precursor concentrations (94(1)% yield). For the surfactant‐assisted synthesis, 50 g citric acid was introduced into SnCl_2_ solution with no addition of NaOH and the reaction duration was increased to 24 h.

Materials Characterization and Testing. PXD was performed using a PANalytical X′pert Pro MPD diffractometer in Bragg–Brentano geometry (Cu Kα_1_ radiation, *λ*=1.5406 Å). Rietveld refinement was performed using the GSAS and EXPGUI software packages,^19^ with the previously published SnSe structure as a reference.^20^ Imaging and elemental analysis were performed by SEM (Carl Zeiss Sigma, at 5 and 20 kV respectively) equipped with EDS (Oxford Instruments X‐Max 80). Further imaging and SAED was conducted by TEM (JEOL 2011, operated at 200 kV). The Seebeck coefficient and electrical conductivity of **1** and **2** were measured using a Linseis LSR‐3 instrument from 300–550 K. Pellets were pressed in a graphite die under Ar (uniaxial pressure of ≈60 MPa; 500 °C; 20 min).

## Supporting information

As a service to our authors and readers, this journal provides supporting information supplied by the authors. Such materials are peer reviewed and may be re‐organized for online delivery, but are not copy‐edited or typeset. Technical support issues arising from supporting information (other than missing files) should be addressed to the authors.

Supplementary
