# Supplementary material for: Facile Surfactant‐Free Synthesis of p‐Type SnSe Nanoplates with Exceptional Thermoelectric Power Factors
Source: Angew Chem Int Ed Engl. 2016 Apr 20;55(22):6433–7. doi: 10.1002/anie.201601420 (PMC5074331; doi:10.1002/anie.201601420)
Supplement: Supplementary file 1 — Supplementary [file ANIE-55-6433-s001.pdf]

Angewandte Chemie
Eine Zeitschrift der Gesellschaft Deutscher Chemiker

Supporting Information

Facile Surfactant-Free Synthesis of p-type SnSe Nanoplates with Exceptional Thermoelectric Power Factors

*Guang Han, Srinivas R. Popuri, Heather F. Greer, Jan-Willem G. Bos, Wuzong Zhou, Andrew R. Knox, Andrea Montecucco, Jonathan Siviter, Elena A. Man, Martin Macauley, Douglas J. Paul, Wen-guang Li, Manosh C. Paul, Min Gao, Tracy Sweet, Robert Freer, Feridoon Azough, Hasan Baig, Nazmi Sellami, Tapas K. Mallick, and Duncan H. Gregory**

anie_201601420_sm_miscellaneous_information.pdf

Experimental details

Materials Synthesis. In a typical synthesis of SnSe nanoplates, 100 mmol NaOH (Sigma, 99.99%) and 10 mmol $SnCl_2 \cdot 2H_2O$ (Sigma, 99.99%) were added into 50 ml deionised water within a two-neck round-bottom flask, in order to prepare a transparent Na_2SnO_2 solution ($SnCl_2 + 4\ NaOH \rightarrow Na_2SnO_2 + 2H_2O$). In parallel, 10 mmol Se (Aldrich, 99.5%) and 20 mmol $NaBH_4$ (Alfa, 98%) were added into 50 ml deionised water within a single-neck round-bottom flask, to prepare a transparent NaHSe solution ($2Se + 4NaBH_4 + 7H_2O \rightarrow 2NaHSe + Na_2B_4O_7 + 14H_2\uparrow$). After the Na_2SnO_2 solution was heated to its boiling temperature using an oil bath, the freshly prepared 50 ml NaHSe aqueous solution was injected into the solution, leading to the immediate formation of a black precipitate. The mixture was heated to boiling again, held for 2 h, and allowed to cool to room temperature under Ar (BOC, 99.998%) on a Schlenk line. The products were collected by centrifuge, washed with deionised water and ethanol several times, and dried at 50 ºC for 12 h in air. The concentration of NaOH addition was varied to observe the effect on the synthesis from 0 through 25, 50, 75 and 100 to 150 mmol (corresponding to $NaOH:SnCl_2$ molar ratios of 0, 2.5:1, 5:1, 7.5:1, 10:1 and 15:1, respectively). Due to the excellent controllability and repeatability of the method, scale-up syntheses with six-fold precursor concentrations were performed, i.e. using 600 mmol NaOH, 60 mmol $SnCl_2 \cdot 2H_2O$, and 300 ml NaHSe solution (0.2 mol L^{-1}); the products demonstrated phase purity and morphology identical to the products synthesised at lower precursor concentrations. For the surfactant-assisted synthesis of SnSe nanoparticles, 50 g citric acid (Alfa, 99.5%) were introduced into $SnCl_2$ solution with no addition of NaOH and corresponding reaction duration was increased to 24 h, while the other experimental conditions were unchanged. The synthesised samples used for characterisation and performance evaluation were stored in an Ar-filled MBraun glove box (< 0.5 ppm H_2O, < 0.5 ppm O_2) to avoid possible oxidation.

Materials Characterisation and Performance Evaluation. The crystal structures of the as-prepared samples were investigated by powder X-ray diffraction (PXD), using a PANalytical X'pert Pro

MPD diffractometer in Bragg-Brentano geometry (Cu Kα1 radiation, λ = 1.5406 Å). Diffraction data were collected with a step size of 0.017° over 10° ≤ 2θ ≤ 90° for 1h (phase indexing) or over 10° ≤ 2θ ≤ 100° for 12h (structural refinement). To determine the lattice parameters and crystal structures of the synthesised products, Rietveld refinement against PXD data was performed using the GSAS and EXPGUI software packages,[1] with the previously published SnSe structure as a basis.[2] For Rietveld refinement against PXD data of pellet samples, a shifted Chebyschev polynomial function and the pseudo-Voigt function (profile function 2 in GSAS) were applied to model the background and peak shape respectively. A March-Dollase preferred orientation parameter along (400) was introduced and refined. The scale factor, zero point and background were refined in initial cycles. The unit cell parameters, peak profile parameters and atomic parameters were refined subsequently.

The morphological and chemical characteristics of the synthesised products were investigated by scanning electron microscopy (SEM, Carl Zeiss Sigma, 5 and 20 kV for imaging and chemical analyses, respectively), equipped with energy-dispersive X-ray spectroscopy (EDS, Oxford Instruments X-Max 80). The synthesised powders were spread on a conductive carbon tape that was then mounted on a standard SEM sample stub. Their structural characteristics were characterised by transmission electron microscopy (TEM, JEOL 2011, operated at 200 kV). To prepare the TEM sample, the SnSe powders were dispersed in ethanol by sonication for 30 s to obtain a uniform dispersion. Then 2-5 drops of the suspension was dropped onto a 3 mm diameter holey C-coated Cu TEM grid. As-synthesised SnSe samples were directly mounted in a Fourier transform infrared (FTIR) spectrophotometer (Shimadzu, FTIR-8400S) to obtain FTIR spectra at room temperature. Thermal analysis of the samples was performed using two thermogravimetric (TG) analysers; a Netzsch STA 409 instrument (providing TG and differential thermal analysis (DTA)) located in an Ar-filled MBraun glove box (< 0.1 ppm H_2O, < 0.1 ppm O_2) and a Netzsch STA 449 F1 analyser (providing TG and differential scanning calorimetry (DSC)). These were used for thermal analysis under flowing Ar and air, respectively. Approximately 25 mg of SnSe pellet was heated to 700 °C in

an alumina pan under flowing Ar (60 ml min^{-1}) at a heating rate of 5 ºC min^{-1} in the former case or heated to 1025 °C in an alumina pan under flowing air (50 ml min^{-1}) at a heating rate of 10 ºC min^{-1} in the latter case. For optical bandgap measurement, SnSe powders were spread into a thin uniform layer on a layer of $BaSO_4$ powder, and then their diffuse reflectance UV-Vis (DR-UV-Vis) spectra were measured by UV-Vis spectrophotometer (Shimadzu, UV-2600) within a wavelength range of 400-1300 nm. To measure the electrical performance of SnSe nanostructures, SnSe powder was loaded into a graphite die and hot-pressed into dense pellets (relative density of ~ 95%) at 500 °C for 20 min under Ar protection with a uniaxial pressure of ~60 MPa. The obtained pellets were cut into bars with the dimension of 12 mm x 3 mm x 2 mm, and their Seebeck coefficient and electrical conductivity of the SnSe bars were measured perpendicular to the hot pressing direction using a Linseis LSR-3 instrument under helium atmosphere within a temperature range of 300-550 K. The uncertainty in the measurement of Seebeck coefficient and electrical conductivity is 5%, leading to ~10% uncertainty for the thermoelectric power factor measurement.

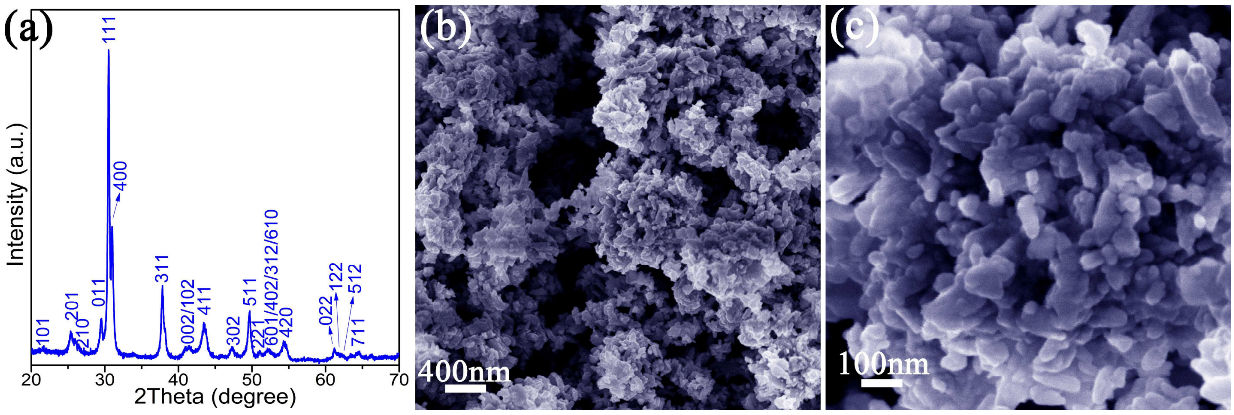

Figure S1. Structural and morphological characterisation of SnSe nanostructures synthesised at room temperature: (a) PXD pattern and (b,c) SEM images at different magnifications.

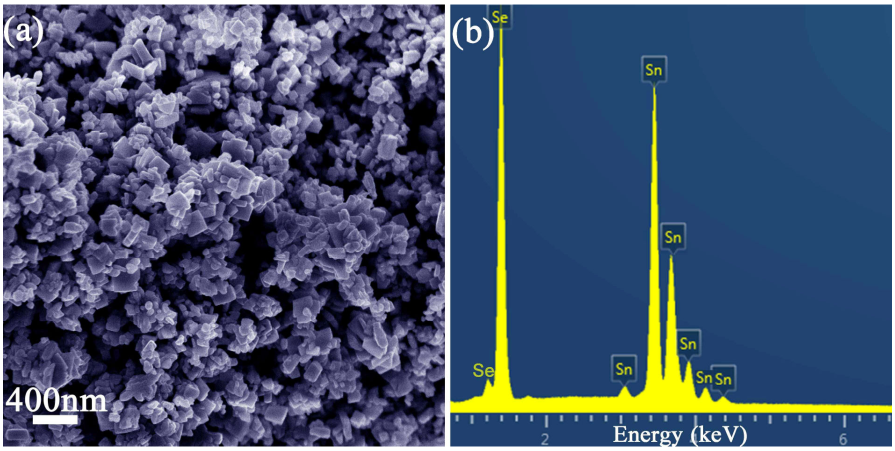

Figure S2. Characterisation of SnSn nanoplates synthesised after 2 h of heating: (a) SEM image and (b) EDS spectrum.

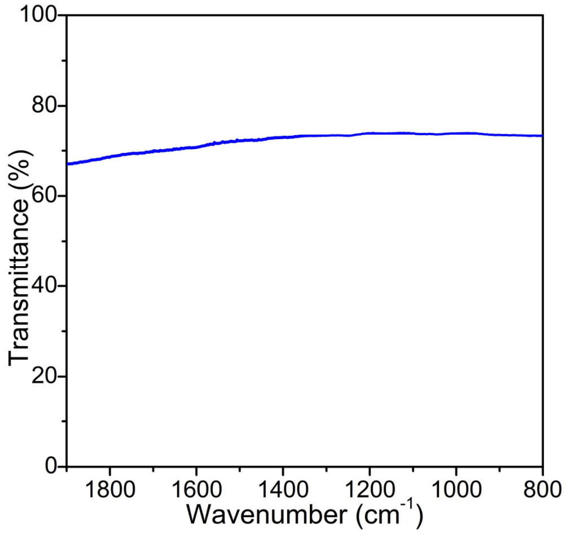

Figure S3. FTIR spectrum of as-synthesised SnSe nanoplates.

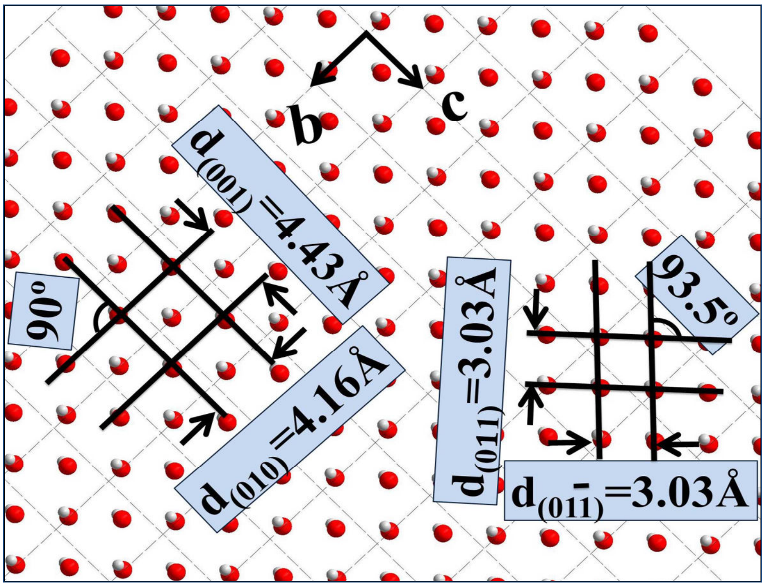

Figure S4. Structure of the *bc* plane in a SnSe nanoplate.

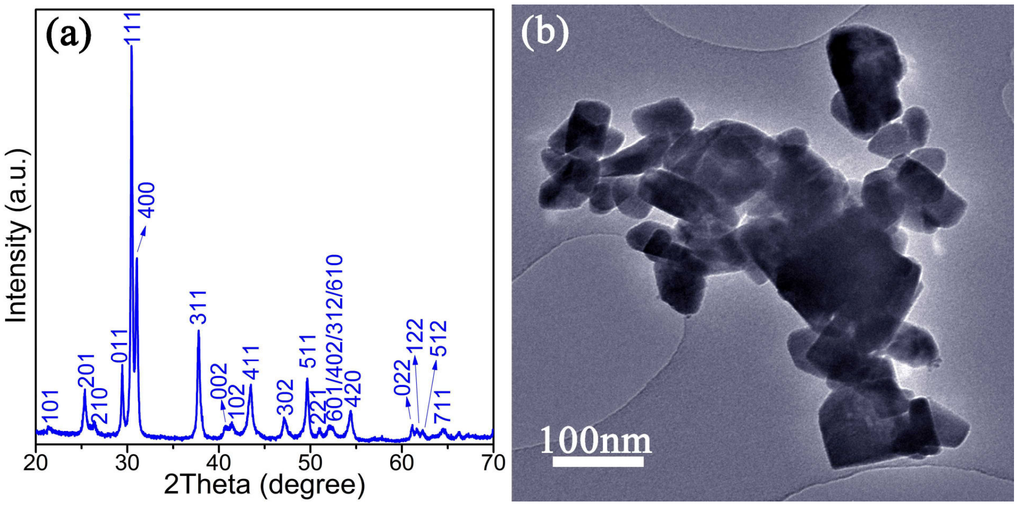

Figure S5. Characterisation of SnSe nanostructures synthesised after 1 min: (a) PXD pattern with all reflections indexed to orthorhombic SnSe; (b) low magnification TEM image.

Effects of changing the NaOH concentration

The NaOH concentration is a crucial synthesis parameter for the controllable synthesis of pure, crystalline SnSe nanostructures with tuneable size. When the NaOH:$SnCl_2$ molar ratio is within the range of 7.5-15:1, a colourless, transparent Na_2SnO_2 precursor solution is formed (Figure S6a); by reacting Na_2SnO_2 with NaHSe, we can controllably synthesise single-phase orthorhombic SnSe (Figure S6b) as discussed previously. In addition, by decreasing the molar ratio from 15:1 to 7.5:1, the mean length/width of the SnSe nanoplates is reduced from ~150 nm (Figure S6c left and Figure S7a) to ~80 nm (Figure S6c right and Figure S7b). On decreasing the NaOH:$SnCl_2$ ratio to 5:1, instead of transformation into Na_2SnO_2 completely, approximately 50% of $SnCl_2$ transferred into black SnO particles with sizes of several micrometres (Figure S6d and Figure S8a-c); the corresponding final products are a mixture of SnSe and Sn (Figure S6e). Given the relatively large particle size of the precipitating SnO, the added NaHSe is likely to react at a relatively slow rate; therefore, SnO may be simultaneously reduced to Sn by Na_2SnO_2 present in the solution.[3] The obtained products are a mixture of nanoparticles and nanoplates (Figure S6f). By contrast, when the NaOH:$SnCl_2$ molar ratio is adjusted to 2.5:1, light yellow $Sn_6(OH)_4O_4$ nanoparticles precipitate (Figure S6g, S8d-f). On addition of NaHSe, nanoparticles approximately 70 nm across of orthorhombic SnSe are obtained (Figure S6h, S6i). Finally, in the case when no NaOH is added, Sn^{2+} is partially hydrolysed generating white, poorly crystalline Sn(OH)Cl nanoparticles (Figure S6j, S8g-i). Although the resulting nanoparticles formed after NaHSe addition can be indexed as single-phase orthorhombic SnSe (Figure S6k, S6l), EDS (Figure S9) indicates the presence of Cl.

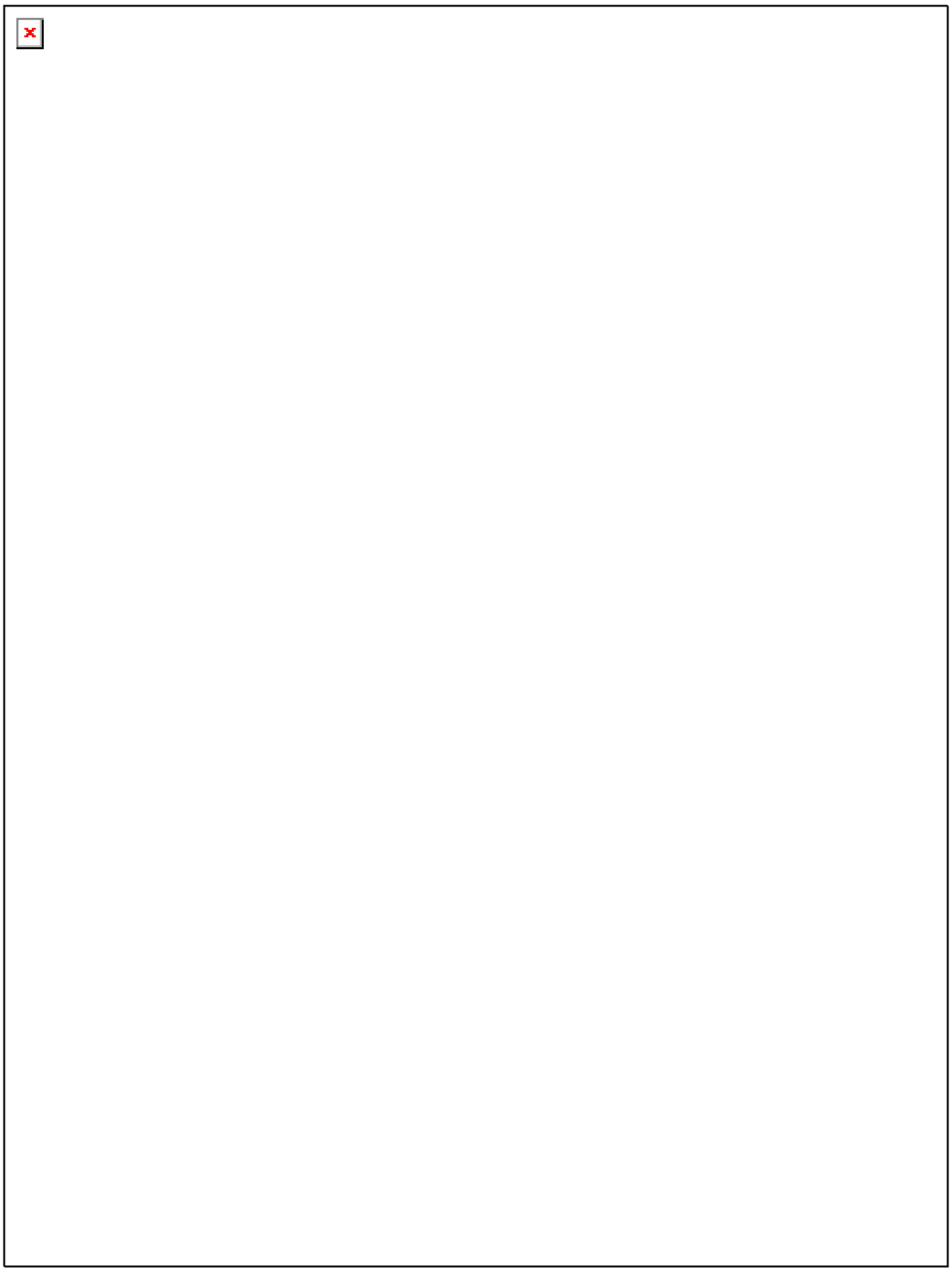

Figure S6. Schematic illustration of the experimental setup and resulting characterisation of SnSe nanostructures synthesised using different NaOH:$SnCl_2$ precursor molar ratios: (a) 7.5:1 – 15:1, (b,c) 7.5:1 and 15:1, (d-f) 5:1, (g-i) 2.5:1 and (j-l) 0 (No NaOH). (a,d,g,j) are the schematic illustrations summarising the apparatus, conditions and colour of the precursor solution; (b,e,h,k) are PXD patterns of the products; (c,f,i,l) are SEM images. 10 mmol and 50 ml respectively of $SnCl_2 \cdot 2H_2O$ and NaHSe aqueous solution were used in each case.

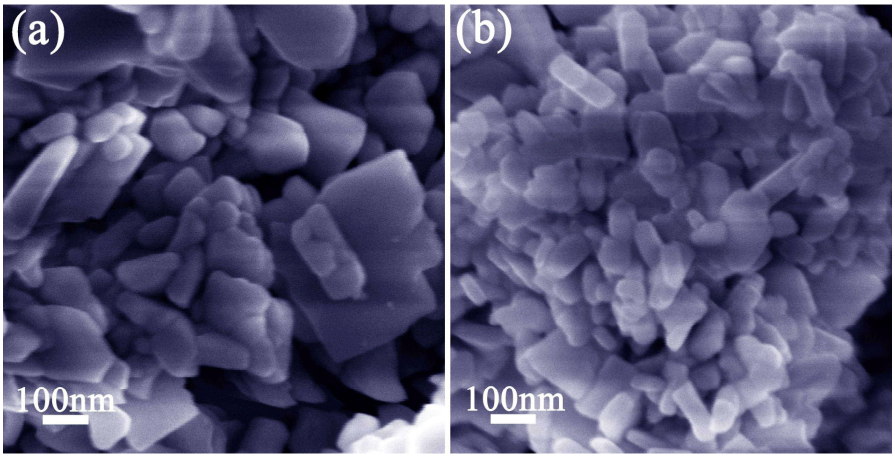

Figure S7. SEM images of SnSe nanostructures synthesised with different NaOH:$SnCl_2$ precursor molar ratios: (a) 15:1, (b) 7.5:1.

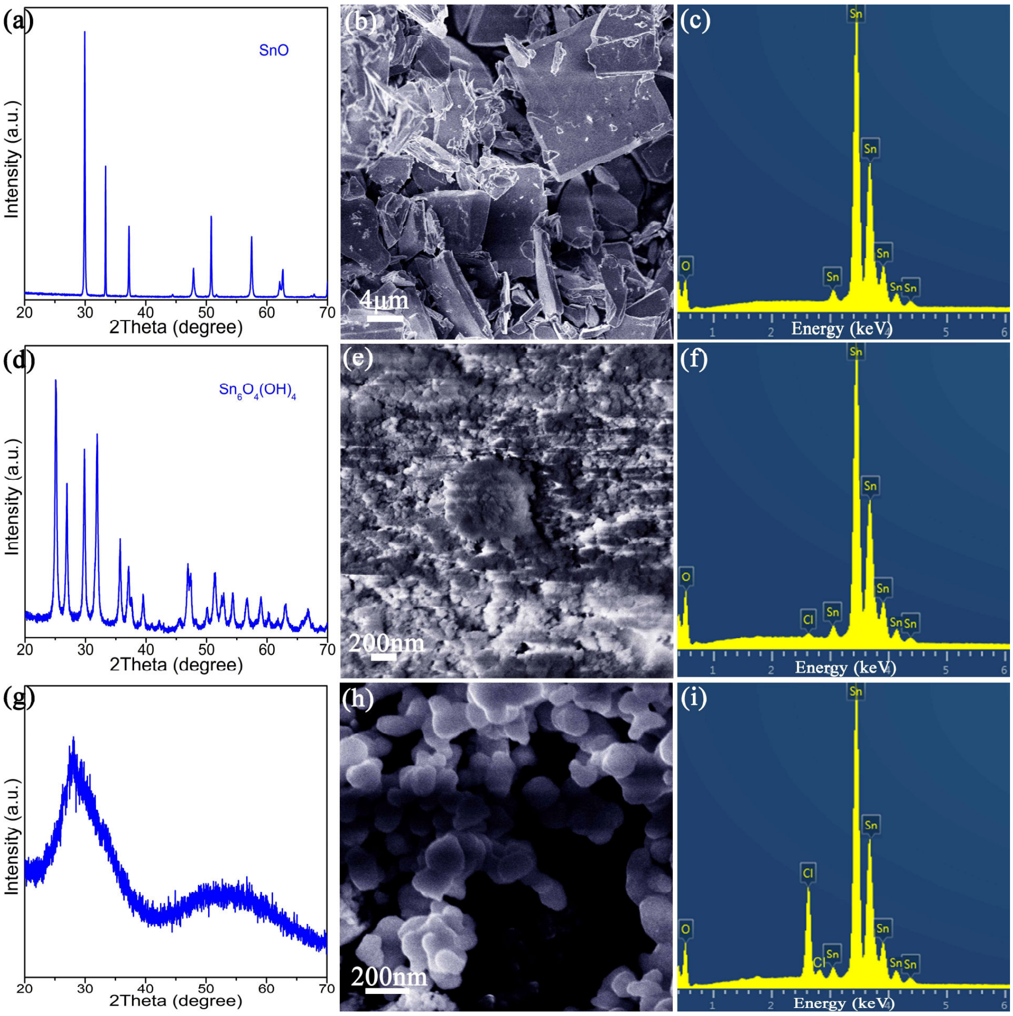

Figure S8. PXD patterns, SEM images and EDS spectra of the various tin-containing precursors formed from different NaOH:$SnCl_2$ molar ratios: (a-c) 5:1 yielding SnO, (d-f) 2.5:1 yielding $Sn_6O_4(OH)_4$ and (g-i) 0 yielding Sn(OH)Cl.

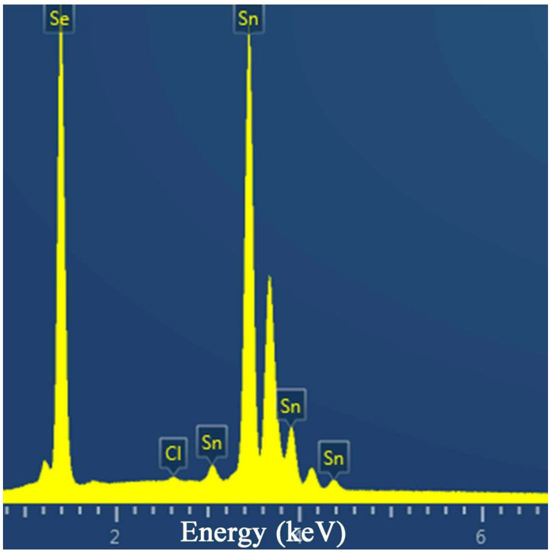

Figure S9. EDS spectrum of SnSe nanostructures synthesised without NaOH addition.

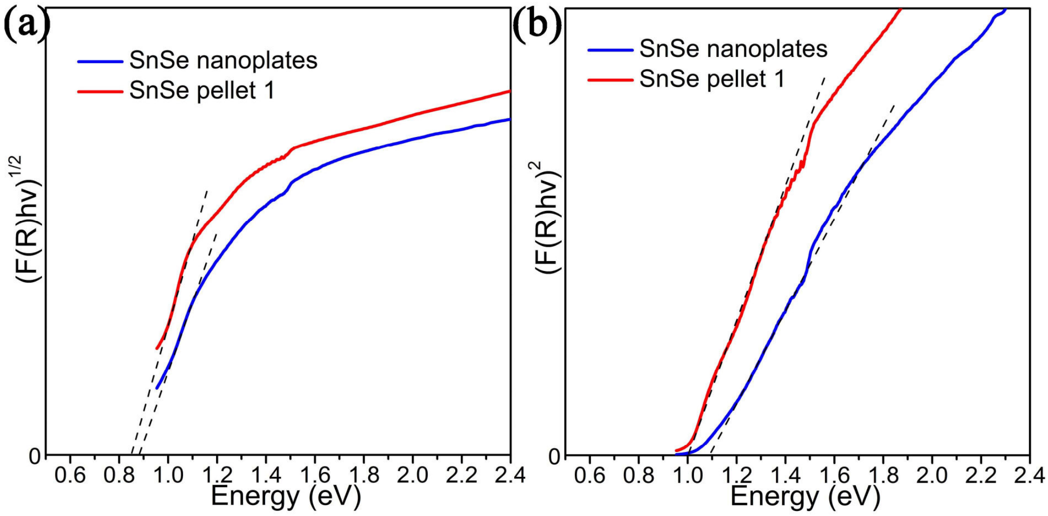

Figure S10. Optical characterisation of SnSe nanoplates and pellet **1**: (a) $[F(R)h\nu]^{1/2}$ *vs* energy plots and (b) $[F(R)h\nu]^2$ *vs* energy plots from DR-UV-Vis spectroscopy data.

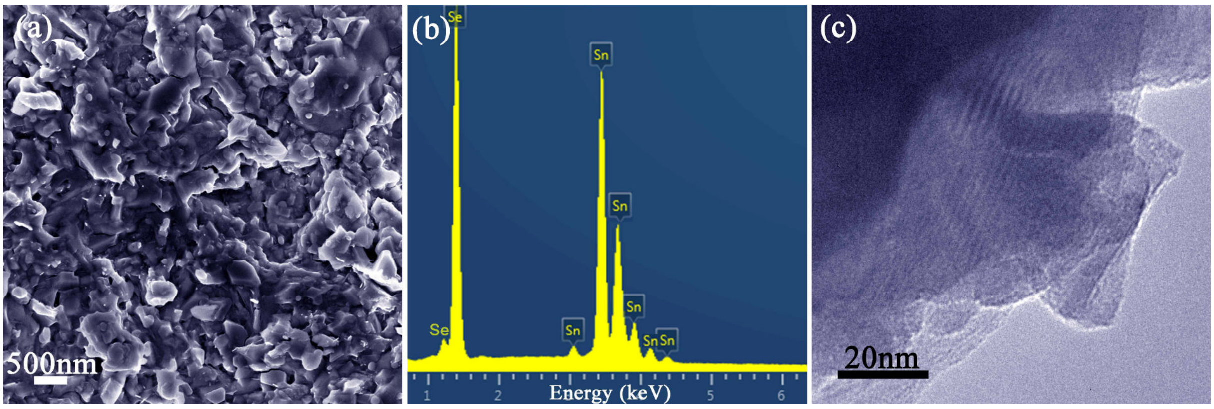

Figure S11. Characterisation of SnSe pellet **1** consolidated from SnSe nanoplates: (a) SEM image; (b) EDS spectrum; (c) TEM image.

Thermal stability under Ar and air.

Figure S12a indicates negligible weight change below 500 ºC followed by a weight loss which can be ascribed to Se sublimation. Resulting PXD and SEM/EDS suggests that SnSe and Sn remain at 700 ºC (the latter is oxidised to SnO_2 during subsequent handling in air; Fig S12c, e, g). Figure S12b reveals negligible weight change below 580 ºC, but a weight gain of ~2.75 wt.% between 580 and 818.5 ºC. This weight gain is attributed to both the oxidation of the SnSe pellet and the sublimation of Se,[4] probably involving the reaction 4 SnSe + 4 O_2 → 2 SnO_2 + 2$SnSeO_2$ + 0.65 Se + 1.35 Se ↑, the theoretical weight gain of which is 2.7 wt%. Between 818.5-1025 ºC, the pellet experienced multi-step sublimation of Se resulting in a total weight loss of ~24.57 wt% during the whole heating process, corresponding to the overall reaction of SnSe + O_2 → SnO_2 + Se ↑. PXD and EDS confirm that the final product after heating in air at 1025 ºC is SnO_2 (Fig S12d, f, h).

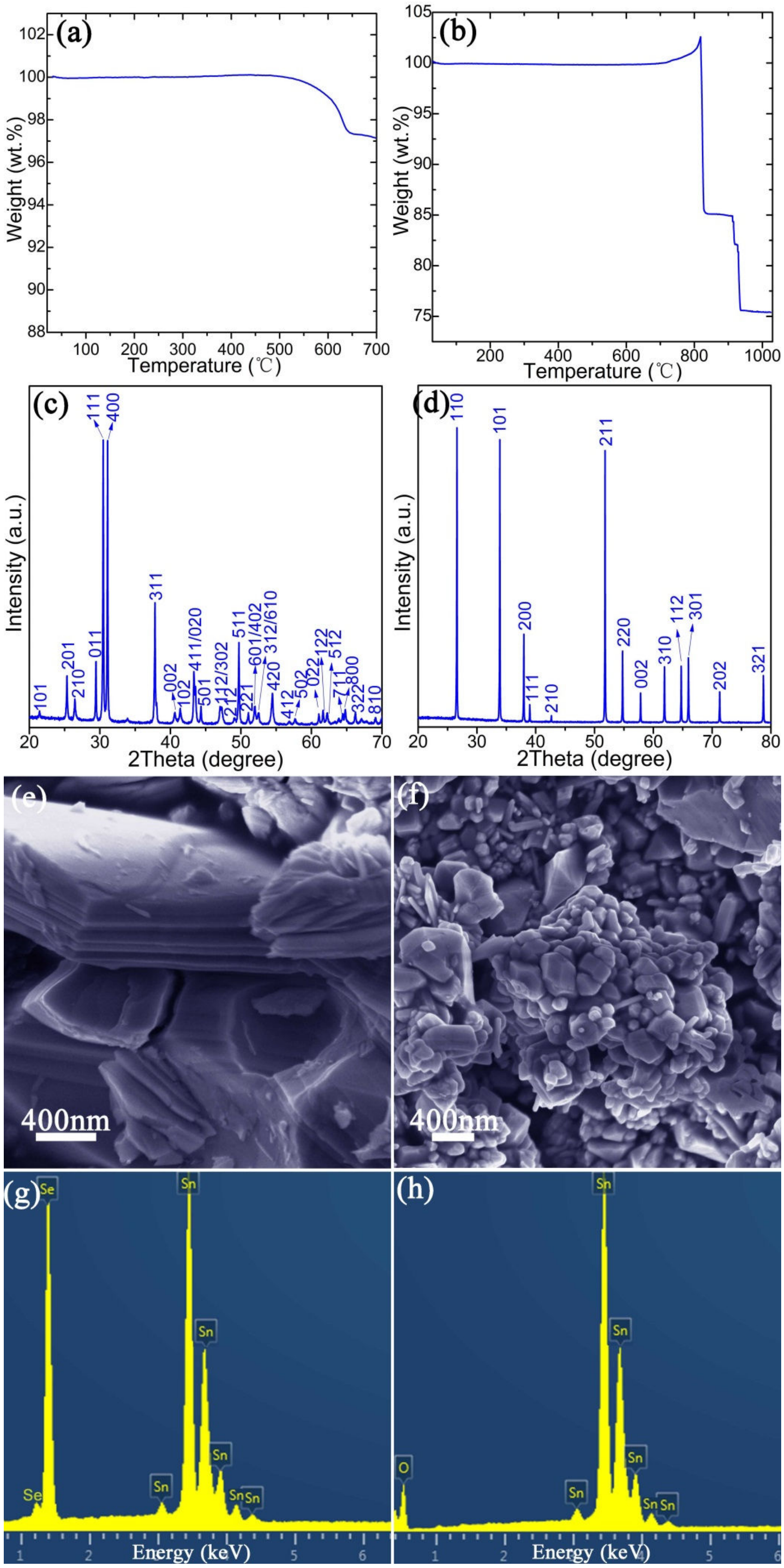

Figure S12. TG profiles of SnSe pellet **1** consolidated from SnSe nanoplates tested under flowing (a) Ar and (b) air; PXD patterns of crushed samples of **1** after TGA under flowing: (c) Ar up to 700 °C and (d) air up to 1025 °C. SEM images of crushed samples of **1** after TGA: (e) under Ar up to

700 °C and (f) in air up to 1025 °C; EDS spectra of (g) the sample imaged in (e) and (h) the sample imaged in (f).

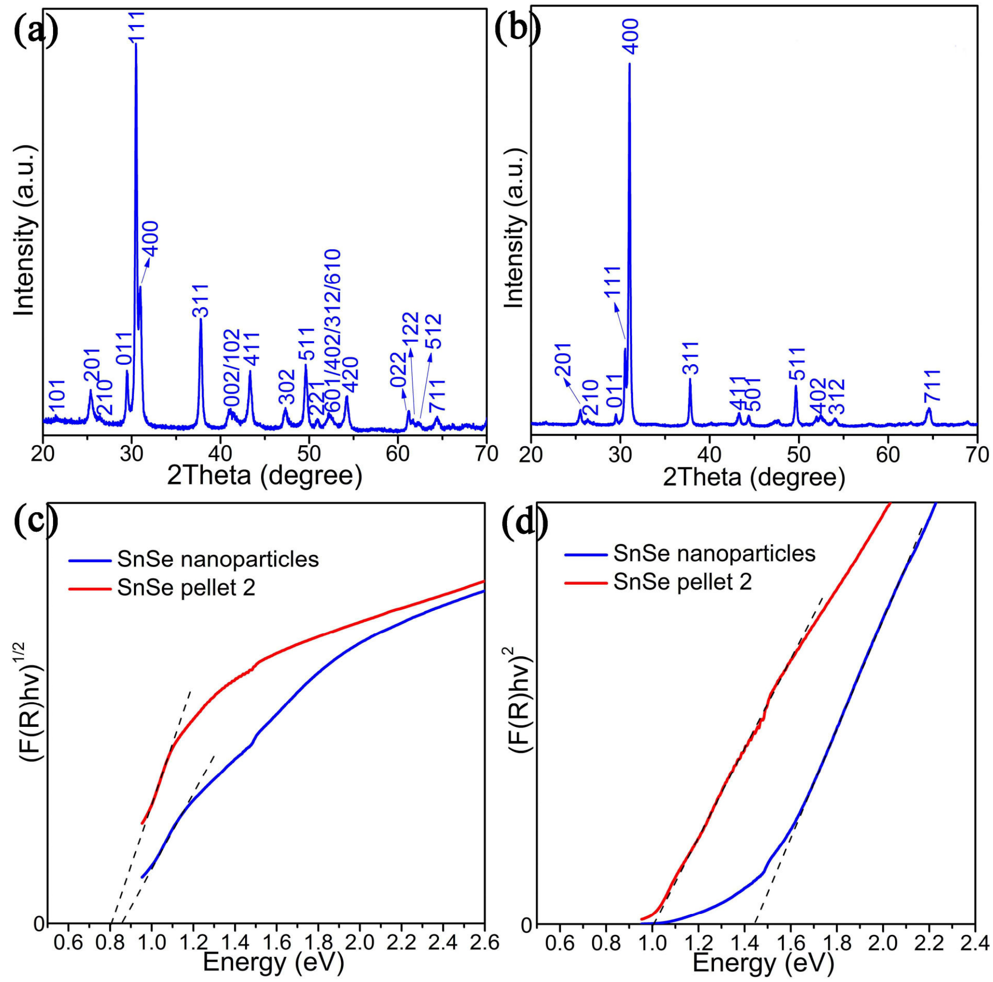

Figure S13. Characterisation of SnSe nanoparticles synthesised by citric acid-assisted synthesis and corresponding hot-pressed SnSe pellet **2**: PXD patterns of the SnSe (a) nanoparticles and (b) pellet; (c) $[F(R)h\nu]^{1/2}$ *vs* energy plots and (d) $[F(R)h\nu]^2$ *vs* energy plots of nanoparticles and pellet **2**.

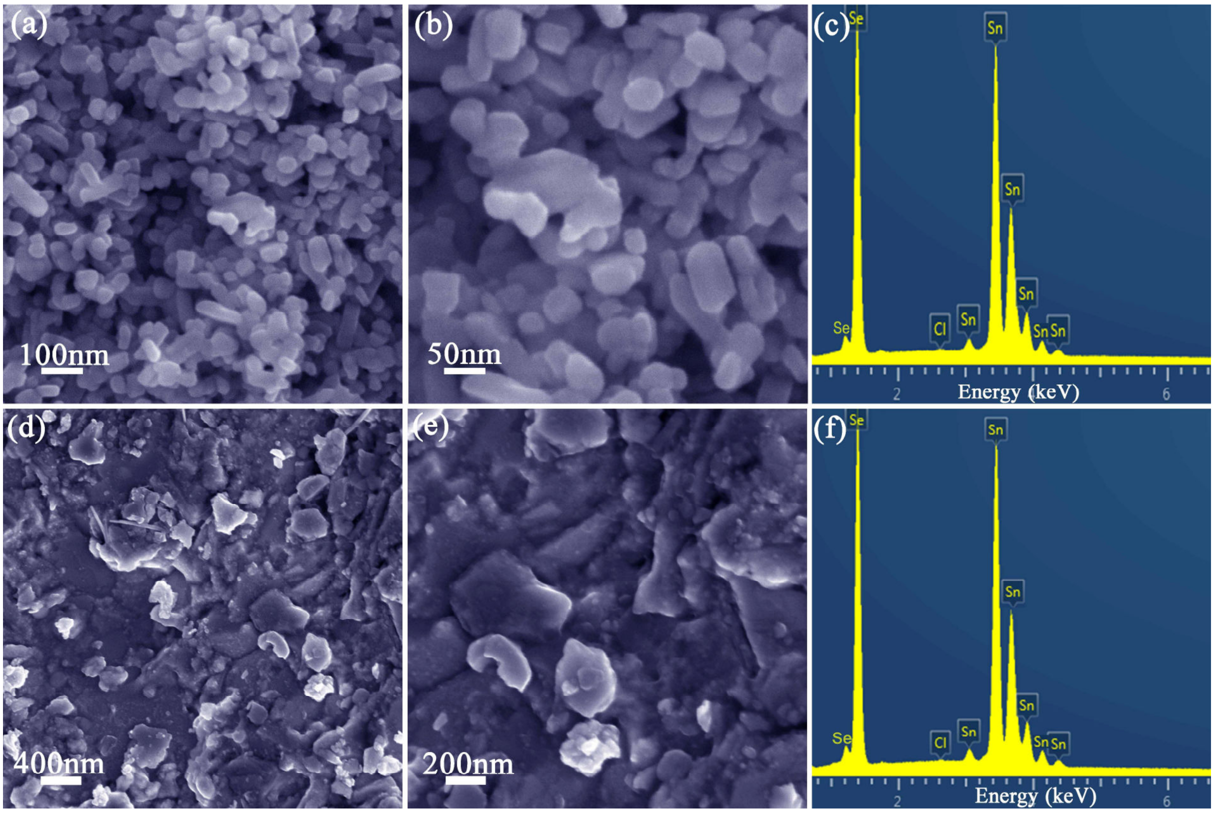

Figure S14. SEM characterisation of SnSe nanoparticles synthesised by citric acid-assisted synthesis and corresponding hot-pressed SnSe pellet **2**: SEM images of the SnSe (a,b) nanoparticles and (d,e) pellet, and EDS spectra of the SnSe (c) nanoparticles and (f) pellet.

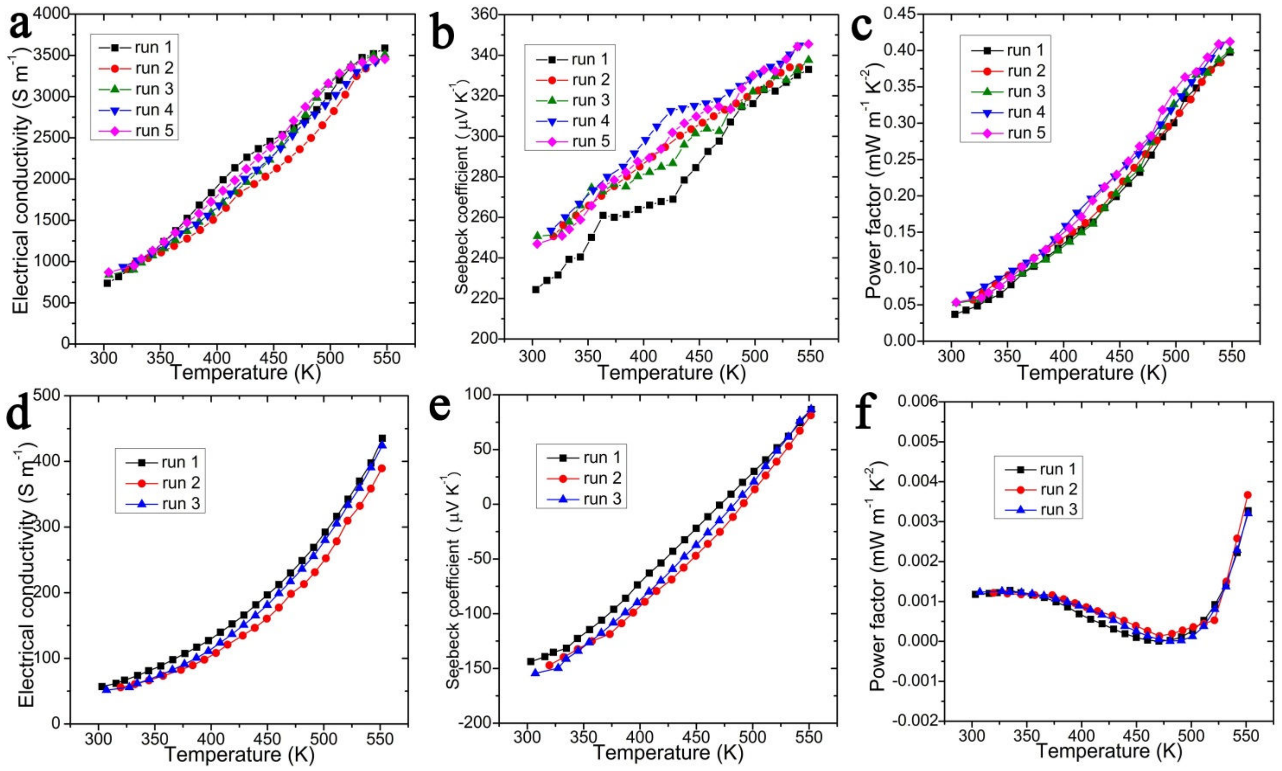

Figure S15. Repeated electrical property measurements (electrical conductivity, Seebeck coefficient, and power factors respectively) of SnSe pellets consolidated from: (a-c) SnSe surfactant-free nanoplates (**1**), and (d-f) SnSe nanoparticles synthesised via the citric acid-assisted method (**2**).

Table S1 Crystallographic data for SnSe nanoplates synthesised after 2 h heating

Chemical Formula	SnSe
Crystal System	Orthorhombic
Space Group	*Pnma*
a (Å)	11.5156(5)
b (Å)	4.1571(2)
c (Å)	4.4302(3)
Volume (Å^3)	212.08(2)
Z	4
Formula Weight (g mol^{-1})	197.65
Calculated density (g cm^{-3})	6.190
R_{wp}	0.0864
R_p	0.0625
χ^2	4.162

Table S2 Atomic parameters for SnSe nanoplates synthesised after 2 h heating

Atom	Wyckoff symbol	x	y	z	100*U_{iso} (Å^2)	Occupancy
Sn	4c	0.12027(12)	0.25000	0.09806(27)	4.75(5)	1
Se	4c	0.35772(17)	0.25000	0.01166(34)	3.14(6)	1

Table S3 Crystallographic data for the SnSe pellet consolidated from SnSe nanoplates **1**

Chemical Formula	SnSe
Crystal System	Orthorhombic
Space Group	*Pnma*
a (Å)	11.5091(3)
b (Å)	4.1601(2)
c (Å)	4.4310(2)
Volume (Å^3)	212.15(2)
Z	4
Formula Weight (g mol^{-1})	197.65
Calculated density (g cm^{-3})	6.188
R_{wp}	0.1197
R_p	0.0829
χ^2	6.521

Table S4 Atomic parameters for **1**

Atom	Wyckoff symbol	x	y	z	100*U_{iso} (Å^2)	Occupancy
Sn	4c	0.11918(10)	0.25000	0.10166(45)	2.36(6)	1
Se	4c	0.35557(14)	0.25000	0.01220(66)	2.82(9)	1

References:

[1] a) A. C. Larson, R. B. Von Dreele, General Structure Analysis System (GSAS); Los Alamos National Laboratory Report LAUR 86-748; Los Alamos National Laboratory, 1994; b) B. H. Toby, *J. Appl. Crystallogr.* 2001, *34*, 210-213.

[2] A. S. Avilov, R. M. Imamov, S. N. Navasardyan, *Kristallografiya* 1979, *24*, 874-875.

[3] A. K. Sinha, A. Sil, A. K. Sasmal, M. Pradhan, T. Pal, *New J. Chem.* 2015, *39*, 1685-1690.

[4] S. Badrinarayanan, A. B. Mandale, V. G. Gunjikar, A. P. B. Sinha, *J. Mater. Sci.* 1986, *21*, 3333-3338.
